# Navigating complexity of the medication management system within the home setting—a functional resonance analysis method (FRAM) analysis of people with dementia and their carers’ experiences

**DOI:** 10.1093/ageing/afae058

**Published:** 2024-03-24

**Authors:** Rosemary Lim, Mais Iflaifel, Zahra A L Qamariat, Clare Crowley, Taniya Sharmeen

**Affiliations:** Reading School of Pharmacy, University of Reading, Reading, Berkshire, UK; Faculty of Life Sciences & Medicine, Department of Pharmacy, King’s College London, London, UK; Pharmaceutical Affairs, Critical Care, Dammam Medical Complex, Eastern Health Cluster, Dammam, Saudi Arabia; Reading School of Pharmacy, University of Reading, Reading, Berkshire, UK; Nuffield Department of Medicine, University of Oxford, Oxford, UK

**Keywords:** dementia, informal carers, medication management, qualitative research, functional resonance analysis method, older people

## Abstract

**Background:**

There is a strong imperative to support people with dementia to live independently in their homes for as long as possible. A starting point is to understand how they manage medications on a daily basis.

**Aim:**

To understand how people with dementia and their informal carers manage medications within the home setting to inform the identification of opportunities to improve medication management.

**Methods:**

A qualitative study using the Functional Resonance Analysis Method (FRAM). Interview data with people with dementia and informal carers were analysed to (i) Identify and describe key functions, (ii) identify and describe variability in performing key functions, and its potential consequences and (iii) map performance variability to Resilient Healthcare capacities.

**Results and discussion:**

A FRAM model was developed and consisted of 14 interdependent key functions. The interdependent nature of functions, and the different nature and sources of variability in how each key function was performed highlighted the level of complexity of the medication management system within the home setting. The medication system was managed almost entirely by the person with dementia and/or their informal carers. This shows the lack of system-level controls to support the safe functioning of the medication management system in the home setting.

**Conclusion:**

Future work will develop a comprehensive FRAM model that includes the perspectives of health and social care professionals and those from the third sectors to underpin the development of a range of system recommendations to strengthen resilience in the medication management system within the home setting.

## Key Points

- A representation of the medication management system from the perspective of people with dementia and informal carers, within the home setting, was conceptualised as a complex system and developed using a systems modelling tool called Functional Resonance Analysis Method (FRAM).
- The FRAM model consisted of 14 interdependent key functions, each of which was performed in different ways.
- The interdependent nature of key functions, and the different nature and sources of variability in how each key function was performed highlighted the level of complexity of the medication management system within the home setting.
- The medication system was managed almost entirely by the person with dementia and/or their informal carers.
- There is a lack of systemic controls to support the safe functioning of the medication management system in the home setting.

## Background

Dementia is a chronic and progressive syndrome of cognitive impairment that causes a decline in daily functioning by affecting memory, behaviour and cognitive abilities of a person [1]. Dementia is caused by various conditions affecting the brain. The most prevalent type of dementia is Alzheimer’s disease, which accounts for 60–70% of dementia cases [2]. Globally, around 10 million people develop dementia each year [3] and its prevalence is expected to nearly double from 35.6 million cases in 2010 to 65.7 million by 2030 [4]. This increase will have profound social and financial consequences, impacting the health and emotional lives of individuals, families and society at large [3, 5].

There are more than 850,000 people living with dementia in the UK [6] and around 61% of people with dementia live at home supported by around 670,000 family carers [7]. More than 90% of people with dementia live with at least one other health condition [8] and with no cure for dementia, medication plays a central role in managing dementia symptoms [9] and treatment of other health conditions while also offering hope. Managing medication in the home is complex for people with dementia and family carers [10] and dominates their daily lives [11]. Due to impairments in cognition and communication, people with dementia may find medication regimens difficult to manage [12] and medication adherence rates can range from 17 to 100% [13]. People with dementia are also three times more likely to be hospitalised due to medication misadventure and when discharged [14], they have a 2-to-3-fold increased risk of taking 30% less or 20% more of their prescribed medication [15].

Problems associated with managing medications within the home setting such as medication errors and frequent hospitalisations, can be a major trigger for admission to a care home. There are trade-offs that have to be considered; moving out of their own home to a care home can cause distress to people with dementia but can possibly reduce the risks of medication problems and carer stress. Changes in routine and environment is problematic for the person with dementia who wants to maintain their independence. Other implications for care such as the variable levels of care provided in care homes and the cost implications at a personal, family and societal level such as the local government-funded care also needs to be considered. There is therefore a strong imperative to support people with dementia to live independently in their homes for as long as possible. One step towards healthy living with dementia is to support people with dementia and/or their informal carers manage medications safely within the home.

The support provided to people with dementia and/or their informal carers needs to be meaningful and underpinned by the realities of the complexity of work that people with dementia and their informal carers do on a daily basis, managing medications. The work of managing medications should therefore be viewed as a complex system, where interdependent parts of the medication system interact in a dynamic way to produce outcomes. Resilient Healthcare theory was therefore used to underpin this study. Resilient Healthcare theory focuses on understanding everyday work (both successes and failures) and the adaptations made (resulting in variable performance) to gain an understanding of the complexity of a work system [16]. Crucially, resilient healthcare is used as means moving away from a linear understanding of complex work and focusing solely on errors [16] to developing a resilient medication management system. An increasing body of research applying resilient healthcare has demonstrated promising results in understanding the complexity of work and in identifying target areas for healthcare interventions for example in hospital, transitions across care settings and care homes [17].

### Study aim

The aim of the study was to understand how people with dementia and their informal carers manage medications within the home setting to inform the identification of opportunities to improve medication management.

## Method

### Study design

**Figure 1:**
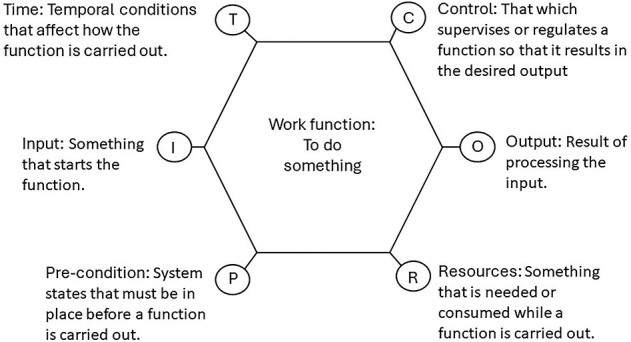
Explanation of the FRAM six aspects, adapted from Hollnagel (2014) [24].

An interpretivist philosophical paradigm [18] and Resilient Healthcare as the explanatory theory were used to underpin this qualitative study using the Functional Resonance Analysis Method (FRAM). FRAM is an increasingly widely used method to represent complex work [19] in a wide range of safety-critical industries [20] including healthcare [21–23]. FRAM decomposes the system into functions, the aim being to move away from ‘what a system is’ to ‘what it does’. It allows the analysis of work functions to produce a model or representation of how work is usually done [14]. Figure 1 shows how a work function, that could be human, technological or organisational, is described. Six aspects are described for any given function; input, output, precondition, resource, control and time. Each function is linked or coupled to another function via their aspects. For example, the input for a function serves as an output for another function. The focus is on understanding the relationship(s) and interdependencies between functions that gives rise to the complexity of work systems.

### Setting and sample

Data collected from a previous study [11] conducted by two of the authors were used in this study.

Data source 1: How do people with dementia make sense of their medications? An Interpretative Phenomenological Analysis study [11].

The methods used for collecting the data are described in a published paper [11]. Briefly, participants were recruited from the Berkshire Healthcare NHS Foundation Trust Research Interested List. This was a list of volunteers who lived with dementia and caregivers who were interested in taking part in dementia research. Those who met the inclusion criteria (see Box 1 in [11]) were approached, recruited and consented by trained researchers at the Berkshire Memory and Cognition Research Centre. Twelve people with dementia consented to take part. Photo elicitation was used to collect data. They were loaned a digital camera and asked to take pictures of objects and places that they viewed to be related to their medication or medication-taking over a two-day period. These pictures were then used as cues in a subsequent in-depth interview conducted by TS with the person with dementia that took place in their own home.

Data source 2: Interviews with informal carers of people with dementia.

Semi-structured interviews were conducted with informal carers of people with dementia.

Participants were recruited from the Berkshire Healthcare NHS Foundation Trust Research Interested List. This was a list of volunteers who lived with dementia and caregivers who were interested in taking part in dementia research. The inclusion criteria were anyone providing any form of help relating to medication use to a relative or relation formally diagnosed with Alzheimer’s Disease or mixed dementia with an Alzheimer’s Disease component. Trained researchers at the Berkshire Memory and Cognition Research Centre identified, recruited and consented participants who met the inclusion criteria. A total of 14 participants consented to be interviewed. Interviews were conducted at a location that suited the participants. TS used a topic guide focused on eliciting participants’ perceptions/views on broad topics specific to medication and medication-taking, experiences relating to their specific role in medication management (e.g. obtaining medication, storage of medication etc.) and strategies used to help the person with dementia manage medications.

### Data analysis

All interview transcripts were analysed as a single body of data set and this process involved:

#### Identify and describe key functions

Interview transcripts from data sources 1 and 2 were coded inductively by three members of the research team, independently, using Microsoft Word. Codes were categorised into clusters with similar ideas e.g. monitor effect of medication, collect medication. The ideas contained in these clusters were discussed amongst the research team to develop initial and then final themes that correspond to key functions in a medication management system. These key functions were then populated onto the FRAM model visualiser [25]. The six aspects for each key function were identified via iterative discussions amongst the research team, drawn from the codes developed as part of the inductive coding of each interview transcript. As an additional sense-checking step, the FRAM model was discussed and finalised with an additional researcher, who was not involved in the initial development process but has experience with FRAM and qualitative research and familiarised herself with both data sources.

#### Identify and describe variability in performing key functions, and its potential consequences

Discussions that took place to develop the FRAM model involved the identification and descriptions of how each key function was performed differently as described by the study participants. Using the FRAM model as a working framework, the research team further analysed the potential consequences of variability to how key functions could be performed.

#### Mapping performance variability to resilient healthcare capacities

To identify potential opportunities to strengthen resilience, variability in performing key functions were mapped to each of the four Resilient Healthcare capacities as described by Hollnagel 2012 [18]; learn, respond, monitor and anticipate.

### Ethics

The study from which data sources were used in this present study received ethical approvals from the United Kingdom National Health Service Health Research Authority (IRAS ID 200310), the English South East Coast—Surrey Research Ethics Committee (reference: 16/LO/1574) and the University of Reading Research Ethics Committee (reference: 16/57). The study was conducted in accordance with the relevant ethical guidelines as set out by the ethics committees. All participants provided written informed consent prior to participation in the study. Participants were informed prior to and during the consent-taking process that their participation was voluntary and that they were free to withdraw from the study should they wish to without prejudice. All participants consented for data collected to be used as part of other University of Reading research studies.

## Results

### FRAM model of the medication management system

**Figure 2:**
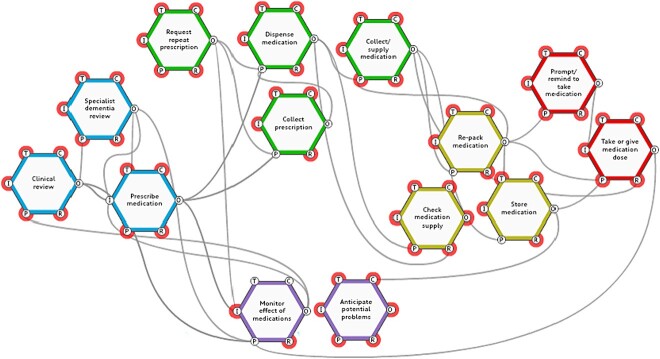
FRAM model of the medication management system for people with dementia within the home setting.

The boundary of the model included any function related to the use of medication in the home setting that involved the person with dementia and/or their informal carer post-diagnosis of dementia. A total of 14 key functions were identified (see Figure 2). Background functions (those that only provide ‘input’ to the key functions) are not represented in Figure 2. The intention is to highlight the key functions that represent the work of the person with dementia and/or their informal carers. The 14 key functions are grouped into five areas of functional activity:

- Clinical review and treatment (blue hexagons): clinical review, specialist dementia review, prescribe medication
- Obtain medication (green hexagons): request repeat prescription, collect prescription, dispense medication, collect/supply medication
- Organise medication (yellow hexagons): store medication, re-pack medication, check medication supply
- Take medication (red hexagons): take/give medication dose, prompt/remind to take medication
- Monitor condition (purple hexagons): monitor effects of medications, anticipate potential problems

There were many-to-many interactions between and amongst the functions, showing the interdependent nature of the functions. For example, the function ‘take or give medication’ is preconditioned on medication being stored in a specific location (i.e. the function ‘store medication’) and/or repacked into another container such as a compliance aid (i.e. the function ‘repack medication’) and the person with dementia and/or the carer having the knowledge and skills in giving or taking medication as prescribed. Each of the functions ‘store medication’ and ‘re-pack medication’ are linked to other functions in the medication system. To ‘take or give medication’, the person with dementia and/or carer must also be present along with the medication itself and any related tools that they use e.g. visual reminders, diaries, list of medications to take (i.e. the resources required). These resources are linked to the function ‘prompt/remind to take medication’ and ‘collect medication supply’. There is also a time element to this function in terms of the time taken to take or give medication, and also the specific period of time during the day where medications were indicated. The output of the function ‘take or give medication’ then triggers the next function to ‘monitor the effects of medication’.

### Variability in performing key functions, and its potential consequences

People with dementia and/or informal carers described variability in how functions relating to managing medications were performed. Table 1 shows examples of functional variability, how these arose and the potential consequences of these variabilities on the medication management system. Where describing a function e.g. prescribe medication, varied tensions and consequences were also reported e.g. delay in treatment or escalation to the media when their preference for a specific dementia medication was not prescribed. The nature of the variability differed ranging from those that were more apparent e.g. an additional or missing medication to subtle changes in the signs and symptoms presented by people with dementia, influencing the threshold for seeking further treatment. The sources of variability were also varied, from those originating from the circumstances of the person with dementia and/or their carer e.g. a planned holiday or an acute illness, to those situated within the wider system e.g. varying types of community pharmacy systems, repeat prescription system or length of time in obtaining treatment, where the person with dementia and/or carer have little to no control of.

**Table 1:** Descriptions and explanations of functional variabilities, their potential consequences on the medication management system and mapping of functional variabilities to resilient healthcare capacities (in *bold and italics*)

Function	Manifestation of variability: what was described?	Tensions and uncertain performance conditions: what are the reasons for this variability?	Functional coupling: what are the potential consequences of this variability?
Clinical review	clinical judgement and experiences of treating people presenting with dementia symptoms implementation of local and National Institute of Health and Care Excellence (NICE) guidelines to diagnose dementia	presentation of signs and symptoms from a person with dementia interpretation of test results	refer or not for specialist dementia review
Specialist dementia review	clinical judgement and experiences of treating people presenting with dementia symptoms implementation of local and National Institute of Health and Care Excellence (NICE) guidelines to diagnose dementia	presentation of signs and symptoms from a person with dementia interpretation of tests and investigation results	no/wrong diagnosis treatment prescribed treatment not prescribed delayed prescribing of treatment
Prescribe medication (for dementia and other conditions)	clinical judgement and experiences of treating people presenting with dementia symptoms, on the part of the prescriber. implementation of local and National Institute of Health and Care Excellence (NICE) guidelines for dementia treatment	presentation of signs and symptoms from a person with dementia willingness of the person with dementia and carer to take medication	treatment prescribed treatment delayed treatment not prescribed escalation by informal carer to the media, and other authorities when a preferred dementia medication was not prescribed
Request repeat prescription (for all prescribed medication)	requests all repeat prescriptions prescriptions not requested prescriptions not requested on time item required not on the repeat prescription request slip ***(anticipate, respond—newly prescribed long-term or short-term medications can often be left off the repeat prescription list. People with dementia and/or carers prefer to request medications themselves to ensure that all their required medication is requested.)*** uses multiple repeat prescription request slips to complete task	different repeat prescription request systems and standard operating procedures amongst the GP surgeries, community pharmacies and patient. The system could be initiated by person with dementia and/or informal carer initiated by the community pharmacy following consent from the person with dementia new/short-term medication	prescriptions ready on time prescriptions not ready for collection triggers different/additional process for doctor to add prescribed medication that was not on the repeat prescription request slip
Collect prescription (for all prescribed medication)	prescription(s) collected as planned prescription(s) not collected prescription(s) not collected on time prescription(s) not ready for collection collects some prescriptions not all required medication are on collected prescriptions ***(anticipate, respond—newly prescribed long-term or short-term medications can often be left off the repeat prescription list, and therefore the prescriptions. People with dementia and/or carers prefer to collect prescriptions themselves to ensure that all their required medication are indeed on the prescription(s).)***	different repeat prescription request systems and standard operating procedures amongst the GP surgeries, community pharmacies and patient. Prescription could be collected and brought to the pharmacy for dispensing by person with dementia and/or informal carer community pharmacy staff GP surgery staff post new/short-term medication	prescription processed further e.g. brought to pharmacy for dispensing delays in prescription collection delays dispensing of medication triggers different/additional process for to add missing medication to the prescription
Dispense medication (for all prescribed medication)	dispenses all medication dispenses part of the medications required dispenses incorrect medication does not dispense required medication dispenses medication in compliance aids	different dispensing and medication supplier systems and standard operating procedures in community pharmacies required medication not on prescription the availability of medication from the manufacturers requirement to supply medication in compliance aids	all medication ready for collection or delivery delay in the collection or delivery of required medication incorrect medication ready for collection or delivery required medication not ready for collection or delivery extra time allocated if medication is to be supplied in compliance aids
Collect medication (all medication)	collect all medication ***(learn, respond – some people with dementia/carers have learnt from the past that there can be problems with the community pharmacy medication delivery system e.g. too late, incomplete medication supply, causing issues with a continuous supply of medication. Some have therefore decided to collect all the medications themselves rather than relying on the community pharmacy to deliver their medications.)*** does not collect all medication only collects some medication collects wrong medication	pharmacy opening times levels of busyness at the pharmacy different community pharmacy medication collection systems and standard operating procedures. early supplies of dementia medication provided by memory clinic prompts to initiate collection or delivery of medication who collects or delivers medication (person with dementia, informal carer, community pharmacy delivery driver)	all required medication collected on time medication collected late required medication not collected wrong medication collected and taken
Re-pack medication (all medication)	does not re-pack medication provided by the pharmacy re-packs into compliance aids, storage containers ***(learn, respond—people with dementia and/or carers knows that taking multiple medications can be confusing and overwhelming. Removing medications from their original packaging and organising them into compliance aids is their way to reduce the level of complexity of taking medications.)***	The need to re-pack medication varies based on dosage form of medication number of prescribed medication how pharmacy supplied medication time and place the person with dementia takes the medication the types of available compliance aids size of medication storage requirements of medications new/short-term medication	entire supply of medication in different containers possible degradation of medication having taken them out from their sealed compartments. additional time taken to organise medication possible errors when re-packing medication into other containers helps person with dementia/carer anticipation when a next supply of medication is needed (visual cues on how many tablets are left) prompt to help person with dementia/carer to take or give medication
Store medication	different storage containers including combination cashbox ***(learn, monitor – an example is of a carer using a combination cashbox as a way to prevent the person with dementia to have free access to medication, to reduce the likelihood of an overdose that had happened in the past.)*** more than one storage container different locations within and outside the house ***(anticipate, respond– some people with dementia and/or carers had developed intricate routines as a prompt to remind the person with dementia to take their medication. For example, medications are placed in a little cup on a red table mat next to the kettle in the kitchen. This is because they would walk into the kitchen first thing in the morning, use a kettle to make tea for breakfast, the time that medication is due. The red table mat is also bright and therefore acts to attract the attention of the person with dementia.)***	clinical status of the person with dementia e.g. ease of remembering where medications are stored, ease of access to medication times where medication is indicated locations where medication is usually taken storage requirements of medications (e.g. fridge) size of the medication storage container new/short-term medication	remembering (or not) where medication was stored or how to store storage of medication linked to another daily activity e.g. medication taken at specific times (morning) and places (kitchen cupboard) prompts the person with dementia to take medication. the use of combination locked boxes prevents the person with dementia to have easy access to medication, and thereby reducing likelihood of an overdose.
Take/give medication	take/give the right doses of all medication, at the right time of day does not take/give all prescribed medication does not take/give prescribed medication at the right time ***(anticipate, respond—there can be times when a medication dose is missed. People with dementia and/or carers know to take the medication as soon as possible if it is not near to the next dose but not to double up the next medication dose if it had been missed entirely.)*** does not take/give the prescribed dose of medication	understanding of the need and/or importance to take/give prescribed medication and how it affects disease progression other daily activities and plans of the person with dementia and/or carer affects commitment to take medications at specific times. new/short-term medication prescribed time of day to take medication—preference to take medication in the morning so they can remember the dose every day. effectiveness of personalised tools/prompts to take medication multiple medications stored in very different places e.g. cupboard, fridge, bathroom changing size, shape and colour of medication	disease progression/remission consternation between dyads about medication taking suspicion as to whether the right medication is given because of changing medication appearances
Prompt/remind to take medication	each person with dementia and carer devised their own plan that consists of different combination of tools as reminders/prompts to take medication. ***(anticipate, respond—for some people with dementia/carers, managing medications is time-consuming, overwhelming and intrusive to their daily living. They anticipate potential issues with medication taking and use tools or a combination of tools as prompts to place time e.g. electronic tablet, telephone, timer, Amazon Alexa Echo, speaking watch and/or compliance aids such as automatic dispenser, daily routine (during breakfast time), carers or at a specific location e.g. on red table mat next to the kettle.)***	the number of medications the prescribed time for taking medication frequency of prescribed medication (daily, weekly, monthly) level of comfort with tools e.g. technology clinical status of person with dementia e.g some people with dementia only take a couple of medications in the morning whilst others are highly dependent on the carer to prompt them storage requirements of medication new/short-term medication	is/is not effective in helping the person with dementia to remember that they need to take medication as intended.
Check medication supply	pill count to check taken doses specific day of week/month to check supply of medication ***(monitor – some people with dementia/carers will conduct a visual check of the compliance aids and/or medication boxes to ensure continuous supply of medication)*** see written notes accompanying medication-taking plans	medication supply cycle new/short term medication frequency of prescribed medication (daily, weekly, monthly)	non-routine medication supply runs out more supply of medication than required
Monitor effects of medication	pill count to check taken doses (not omitted or overdose) ***(monitor – some people with dementia/carers will conduct a visual check of the compliance aids and/or medication boxes to reassure themselves that medication had been taken, or highlight potential issues if there are irregularities.)*** observes new signs and symptoms observes changes in signs and symptoms	subtle changes in signs and symptoms	delays in/does not seek opinion about new/changes in signs and symptoms disease progression
Anticipate potential problems	only responds when problems occur (this can be related to medication supply and/or condition of the person with dementia) ***(respond, monitor, anticipate – acute illness such as an infection can disrupt the routines established by the person with dementia and/or carer. In such cases, there are extra* steps *taken to monitor the health condition of the person with dementia.)*** proactive in identifying potential issues e.g. carer going on holiday, person with dementia going on holiday, changes in treatment, short-term prescription of medication. ***(respond, anticipate—non-routine activities such as a holiday can disrupt the routines established by the person with dementia and/or carer. In such cases, there are extra* steps *taken to ensure that the person with dementia has sufficient medication for the duration of the holiday or if the carer is to be away from the person with dementia, ensuring that there is a trusted person whom the person with dementia can contact.)***	person with dementia’s level of independence/dependence on others e.g. carer other concurrent health conditions and medications taken	disease progression runs out of medication detailed plan to support the person with dementia with medication

### Mapping functional variability to resilient healthcare capacities

Despite the nature and sources of variability, the person with dementia and/or their informal carers developed different ways to undertake relevant functions in the medication system. These different ways of working, presented as descriptions of performance variability of functions in the medication system in Table 1, are mapped to the four key capacities of resilient healthcare, those of respond, learn, monitor and anticipate. The relevant resilient healthcare capacities and an accompanying description are presented in italics in Table 1. It is also interesting to note that some key functions in the medication management system as presented in Figure 2, directly reflect capacities of Resilient Healthcare e.g. anticipate potential problems and monitor effects of medication.

The onus is almost solely placed on the person with dementia/informal carer when managing medication. Some participants discussed their need to ‘control’ the entire medication management process e.g. they request their repeat medication, collect the prescription from the GP surgery, bring the prescription to the community pharmacy for the community pharmacy to dispense the medication and pick up medication from the community pharmacy. They experienced problems relating to one or more key functions in the past e.g. the required medication was not on the prescription, an error to the medication dispensed. These problems had led to other problems e.g. missing medication doses. They learnt from past experiences of that each of these functions can be subjected to variability resulting in negative outcomes. They anticipated possible issues and ‘controlling’ the various functions was their way of responding to uncertainty, and monitoring the process so they can respond promptly and appropriately when issues arise. This is in contrast with others who consented for the community pharmacy to undertake the process of requesting repeat medications through to dispensing medication, thereby removing the responsibility for these functions from the person with dementia and/or informal carer.

Another example is that of a carer using a combination cash box to store the medication of the person with dementia. This was a decision made by the carer following the person with dementia taking an accidental overdose of their medication. Using a combination cash box was the person with dementia/informal carer’s response to the overdose event to take control and prevent a similar event happening in the future. They worked on their own to resolve this medication problem. Other people with dementia and informal carers had developed a good relationship with their community pharmacy and work together, rather than on their own, to resolve issues with medication e.g. an emergency supply when medication runs out.

## Discussion

### Principal findings

To the authors’ knowledge, this study is the first to use FRAM to represent how people with dementia and their informal carers manage medications within the home setting, underpinned by resilient healthcare theory. A total of 14 key functions were identified. These functions were interconnected with each other showing their dependence on one another for their functioning. There was variability in how each of the key functions was performed. The nature and sources of variability also differed; those of the person with dementia and/or their informal carer, or those within the wider system e.g. community pharmacy systems, repeat prescription system, receiving a diagnosis, where the person with dementia and/or informal carer have no direct control over.

The interdependent nature of functions and variability in the nature and sources of variability highlights the level of complexity of the medication management system within the home setting. In addition, the burden of care was on the person with dementia and/or their informal carers; the medication system was managed almost entirely by them. Managing a complex medication system within the home setting requires capacity, resources and confidence from the person with dementia and/or their informal carer, which they may or may not have. The actions or non-actions of the person with dementia and/or their informal carer were therefore critical in the functioning of a dynamic medication management system. Relying heavily on the person with dementia and/or their informal carer to manage the complex medication system suggests a lack of system-level controls, beyond that of the context of their immediate home setting.

### Representation of and implications of complex patient work

The complexity of managing medications by people with dementia and/or their informal carers has been previously documented (examples include [10, 11, 26–28]). Our study adds to this evidence base further by confirming that medication management within the home setting is indeed complex, and should be viewed as a complex system. In addition, our study showed, using the FRAM model, how the complexity of medication management unfolds in terms of the interdependent nature of specific key functions, and the types and sources of variation relating to how a function is performed.

Despite the variable sources of variability, the person with dementia and/or their informal carers had developed ways to adapt or adjust their needs within the context of the wider healthcare system structure. For example, they anticipate problems that may occur when a new short-term medication is prescribed, they work closely with community pharmacies to streamline the medication ordering process or store medication in a combination (locked) to avoid an overdose. These adaptations however, do not always result in intended outcomes. For example, not all the medication required by the person with dementia is always ordered. The person with dementia can also feel disempowered and their sense of self altered with the introduction of certain interventions, as demonstrated by an example of a quote by a person with dementia who was interviewed for the study [11] ‘Am I a simpleton?’

Healthcare professionals are also a key part in the medication management system in the home setting. Their roles and responsibilities such as diagnosis, prescribing, dispensing and monitoring the effect of medications, have a direct impact on the way medication is managed within the home setting. Therefore, there is a need for healthcare professionals who come into direct contact with people with dementia and/or informal carers to understand the level of complexity in managing medications including the nature and sources of variability and its impact on the person with dementia and/or informal carer. Open discussions and constant dialogue with people with dementia and/or informal carers about challenges with medication management, including deprescribing if appropriate, is important to share the burden of everyday care or self-care by the person with dementia. An honest discussion about medication errors that occur within the home setting, without blame and/or removing the independence or autonomy of the person with dementia is also key in uncovering and addressing the need for stronger systemic support to create a resilient medication management system in the home setting.

Adaptations made by the person with dementia and/or their informal carers may resolve a particular issue for a period of time. But the progressive nature of dementia, the variability in how dementia presents on a daily basis (good days and bad days for the person with dementia), the specific and changing needs of informal carers (many of whom also have other health conditions [29]) and the dynamic and changing nature of the healthcare system itself present challenges that may not be easily anticipated (due to the level of complexity). These challenges cannot reasonably be managed or sustained by the person with dementia and/or their informal carers on their own. There is therefore an imperative to consider, from a systems perspective, how the medication management system can be resilient. The medication management system, as represented by the FRAM model in this study can form the basis to identify key areas for system intervention. The focus should be on considering the nature and types of interactions and interdependence between and amongst key functions in this medication system, and use these to underpin the design of systemic interventions with people with dementia and their informal carers.

### Strengths and limitations

A key strength was the inclusion of the voices of people with dementia in the study to represent the medication management system, rather than proxy data sources. The use of photo elicitation as a data collection approach was also novel as it did not rely on the memories of the person with dementia at a single point in time. This study also represents the first system and functional representation of the medication management system from the perspective of people with dementia and/or their informal carers, within the home setting.

The FRAM model may not be exhaustive in terms of its comprehensiveness. Data sources from others involved in the medication management system such as health and social care professionals (e.g. district nurse, social workers, doctors, pharmacists) were not used in the development of the FRAM model. These additional data sources could add to the existing FRAM model. Further information about the detection and anticipation of problems may add further insights to the nature and sources of key function variability such as medication supply issues and the digitisation of health information. Participants were recruited from a single research centre in the UK. There could be different medication systems e.g. repeat medication system, dispensing systems, used in other parts of the country, and therefore possible variations in how key functions can be performed. Thereby, it is possible that the medication management system could increase in complexity. The FRAM model was not checked with people with dementia and/or informal carers due to the time from the interviews and the nature of dementia. There could therefore be adjustments to the FRAM model.

### Future work

The next step is to develop a comprehensive FRAM model that includes the perspectives of other health and social care professionals (particularly community pharmacies in the first instance) and those from the third sectors (e.g. voluntary and community groups and, charities, supporting people with dementia and/or carers). This will underpin a range of system recommendations that can be further developed into solutions to further develop and strengthen resilience in the medication management system within the home setting. The design work will need to be co-produced with people with dementia, informal carers and those working in health, social and third sectors.

For people newly diagnosed with dementia, and their informal carers, an early understanding of the complexity of the medication management system can both be helpful and daunting in equal measure. Sharing such information will need to be carefully considered and another future piece of research can explore how best to communicate the realities of managing medications within the home setting.

## Conclusion

The complexity of the medication management system from the perspective of people with dementia and/or their informal carers, within the home setting, was represented in a FRAM model for the first time. Fourteen interdependent key functions were identified along with descriptions of the variability of how they were performed. The burden of everyday care was on the person with dementia and/or their informal carers and this requires capacity, resources and confidence. There is a lack of systemic controls to support the safe and resilient functioning of the medication management system in the home setting. Future work will involve developing a comprehensive FRAM model that includes the perspectives of other health and social care professionals and those from the third sectors to underpin the development of a range of system recommendations to strengthen resilience in the medication management system within the home setting.
